# Supplementary material for: LTD at amygdalocortical synapses as a novel mechanism for hedonic learning
Source: eLife. 2020 Nov 10;9:e55175. doi: 10.7554/eLife.55175 (PMC7655100; doi:10.7554/eLife.55175)
Supplement: Transparent reporting form [file elife-55175-transrepform.docx]

***eLife’s* transparent reporting form**

We encourage authors to provide detailed information *within their submission* to facilitate the interpretation and replication of experiments. Authors can upload supporting documentation to indicate the use of appropriate reporting guidelines for health-related research (see [EQUATOR Network](http://www.equator-network.org/%20)), life science research (see the [BioSharing Information Resource](https://biosharing.org/)), or the [ARRIVE guidelines](http://www.plosbiology.org/article/info:doi/10.1371/journal.pbio.1000412) for reporting work involving animal research. Where applicable, authors should refer to any relevant reporting standards documents in this form.

If you have any questions, please consult our Journal Policies and/or contact us: [editorial@elifesciences.org](mailto:editorial@elifesciences.org).

**Sample-size estimation**

- You should state whether an appropriate sample size was computed when the study was being designed
- You should state the statistical method of sample size computation and any required assumptions
- If no explicit power analysis was used, you should describe how you decided what sample (replicate) size (number) to use

Please outline where this information can be found within the submission (e.g., sections or figure legends), or explain why this information doesn’t apply to your submission:

No explicit power analysis was used, as the size of the effects could not be estimated prior to obtaining a full data set.

We ensured that every parameter measured could be replicated across animals from different litters. The number of neurons from each animal was comparable to ensure reproducibility and reliability of our findings.

Analysis was performed blind to experimental conditions, to ensure lack of bias.

**Replicates**

- You should report how often each experiment was performed
- You should include a definition of biological versus technical replication
- The data obtained should be provided and sufficient information should be provided to indicate the number of independent biological and/or technical replicates
- If you encountered any outliers, you should describe how these were handled
- Criteria for exclusion/inclusion of data should be clearly stated
- High-throughput sequence data should be uploaded before submission, with a private link for reviewers provided (these are available from both GEO and ArrayExpress)

Please outline where this information can be found within the submission (e.g., sections or figure legends), or explain why this information doesn’t apply to your submission:

Experiments were performed at 2-3 time per week.

Technical replication was performed to ensure reliable and comparable expression of optogenetic constructs across animals. Titration of viral particles and injection volume was performed in 15 animals. Histological verification of injection sites was used to assess injection parameters. In animals in which the injection site was targeted correctly, expression of the construct in terminal fields was used to establish a calibration curve against which all data from other animals would be compared. Once optimal injection parameters were established, they were used to establish criteria for inclusion of electrophysiological data. Only animals for which injection site, and the neuron type and location was verified histologically were included in the study.

The number of animals and neurons used for this study is reported in the text and figure legends. Replicates were obtained from several animals belonging to different litters to ensure reproducibility and reliability of our findings.

For electrophysiological data, basic parameters such as input resistance and series resistance were used to make decisions about inclusion in the analysis. For optogenetic stimulation, we ensured comparisons across preparations with comparable expression of constructs by establishing a calibration curve of expression. Data obtained from animals with construct expression 1 standard deviation higher or lower than the calibration curve were not included in the analysis.

Potential outliers were reanalyzed to verify whether they met inclusion parameters. If they did, they were not excluded from the analysis.

Our study does not contain high throughput sequencing data.

**Statistical reporting**

- Statistical analysis methods should be described and justified
- Raw data should be presented in figures whenever informative to do so (typically when N per group is less than 10)
- For each experiment, you should identify the statistical tests used, exact values of N, definitions of center, methods of multiple test correction, and dispersion and precision measures (e.g., mean, median, SD, SEM, confidence intervals; and, for the major substantive results, a measure of effect size (e.g., Pearson's r, Cohen's d)
- Report exact p-values wherever possible alongside the summary statistics and 95% confidence intervals. These should be reported for all key questions and not only when the p-value is less than 0.05.

Please outline where this information can be found within the submission (e.g., sections or figure legends), or explain why this information doesn’t apply to your submission:

Statistical information can be found in the methods, in the Figure legends and in the Source Data Files. The number of animals, number of neurons and confidence intervals are reported for each data set. Estimation statistics was used to assess significance.

(For large datasets, or papers with a very large number of statistical tests, you may upload a single table file with tests, Ns, etc., with reference to sections in the manuscript.)

**Group allocation**

- Indicate how samples were allocated into experimental groups (in the case of clinical studies, please specify allocation to treatment method); if randomization was used, please also state if restricted randomization was applied
- Indicate if masking was used during group allocation, data collection and/or data analysis

Please outline where this information can be found within the submission (e.g., sections or figure legends), or explain why this information doesn’t apply to your submission:

Specific experiments were designed for each data set.

**Additional data files (“source data”)**

- We encourage you to upload relevant additional data files, such as numerical data that are represented as a graph in a figure, or as a summary table
- Where provided, these should be in the most useful format, and they can be uploaded as “Source data” files linked to a main figure or table
- Include model definition files including the full list of parameters used
- Include code used for data analysis (e.g., R, MatLab)
- Avoid stating that data files are “available upon request”

Please indicate the figures or tables for which source data files have been provided:

Source Data Files are provided for each figure and corresponding supplement. The Source data include information about statistics used for each data set.
